# Replacing Fertilizer with Dried Distillers’ Grains in Stocker Cattle Systems on Southern Great Plains Old World Bluestem, USA

**DOI:** 10.3390/ani13182904

**Published:** 2023-09-13

**Authors:** Brody D. Wallis, Phillip A. Gunter, Gerald W. Horn, Ryan Reuter, Brian Arnall, Jason Warren, Sarah R. Lancaster, Phillip A. Lancaster

**Affiliations:** 1 Department of Animal Science, Oklahoma State University, Stillwater, OK 74075, USA; 2 Department of Plant and Soil Sciences, Oklahoma State University, Stillwater, OK 74075, USA

**Keywords:** economics, greenhouse gas emissions, nitrogen use efficiency

## Abstract

**Simple Summary:**

Nutrient losses from grazing systems are a source of water and air pollution and a cost to producers, as these nutrients must be replaced. Improved management systems are needed in order to reduce nutrient losses. Feeding cattle directly and allowing manure to fertilize the grass improved nutrient use efficiency, but was not always more economical compared to fertilizing the grass directly. Replacing fertilizer with feed supplements is a viable management system to reduce nutrient losses from grazing systems.

**Abstract:**

The objective was to examine the effects of dried distillers’ grains supplementation and fertilization strategies on the cattle performance and resource use efficiency of stocker cattle grazing on Plains Old World bluestem. Over 4 consecutive years, heifers and steers (average n = 239) were randomly assigned to 1 of 4 treatments: (1) low input, low stocking density, and no fertilizer or distillers grains supplementation (LOW); (2) high stocking density and no fertilizer with distillers grains supplementation (DDGS); (3) high stocking density and 90 kg of nitrogen/ha with no distillers grains supplementation (NFERT); (4) high stocking density, 90 kg of nitrogen/ha, and 39 kg of phosphorus/ha with no distillers grains supplementation (NPFERT). Cattle grazed in the pastures from mid-May to mid-September each year, except for 2011, when the experiment ended in July due to lack of forage. Data were analyzed using a linear model with fixed effects of treatment, year, and treatment × year (R software). Nitrogen use efficiency (retained/inputs) was affected by a treatment × year interaction, where LOW had the greatest efficiency in all years and DDGS was greater than NFERT and NPFERT in all years except 2012, with NFERT and NPFERT being not different in all years. The estimated total carbon equivalent emissions were greater for DDGS, NFERT, and NPFERT than LOW, but the carbon footprint (kg CO_2_eq/kg weight gain) was lesser for LOW and DDGS, which were not different, than NFERT and NPFERT, which were also not different. Replacing nitrogen fertilizer with dried distiller’s grains improved the cattle performance and the efficiency of resource use, and could be a viable economic alternative to traditional systems.

## 1. Introduction

In the U.S. southern Great Plains, the grazing of warm-season perennial grasses is a common practice for increasing the weights of growing beef cattle while striving to achieve acceptable body weights and quality for entrance into feedlots. Several species of introduced warm-season forages are grazed throughout the summer months, and many of these introduced species respond well to nitrogen (N) fertilization. Old World bluestem (*Bothriocholoa ischaemum* L.) is one warm-season introduced perennial grass that has been planted in large areas of the southern Great Plains to assist in arresting soil erosion on marginal farmland and as a high-quality summer forage for cattle [1]. The use of N fertilizer is a common practice to increase forage yields in Old World bluestem. Berg [2] reported an almost linear yield response by Old World bluestem to N fertilizer, up to 70 kg N ha^−1^ yr^−1^. This increase in yield would result in greater forage mass and increased stocking rates, resulting in improved ADG, as reported by Ackerman et al. [3].

Dried distillers grains with solubles, a co-product of the ethanol production industry, have proven to be an effective feed source for both grazing and confinement beef cattle programs. The improvement in ADG with distillers grains supplementation to grazing cattle has been widely documented [4]. Distillers grains are typically a cost-effective feed high in energy, protein, and phosphorus (P). 

The importance of managing nutrients in cattle production programs has been accentuated in recent years due to the increased concerns of environmental impact as well as the economic advantage of effectively utilizing these nutrients. Wilkinson and Langdale [5] reported that as N fertilizer application rates increased, retention of N inputs in beef gain decreased on warm-season grasses in the southeastern USA. About 75% of nitrogen, 80% of phosphorus, and 90% of potassium ingested by animals is excreted [6], which results in highly concentrated N, P, and K being recycled by the soil and plants, or lost into water bodies or to the atmosphere. Additionally, N application in the form of fertilizer or animal excreta, especially when the quantity exceeds the plant’s ability to assimilate N, results in nitrate leaching and nitrous oxide (N_2_O) emissions, a potent greenhouse gas with 298 times the global warming potential of carbon dioxide (CO_2_) [7]. Nitrogen losses from the grazing system occur each time N changes form (soil nitrate/ammonia, microbial N, plant N, animal protein) as it cycles within the system. Therefore, N inputs in the form of feed rather than fertilizer could result in greater N recovery in beef cattle grazing programs due to fewer transformations.

There have been documented improvements in weight gain [8] and N use efficiency [9] when replacing fertilizer with distillers grains in grazing systems of smooth bromegrass (*Bromis inermis*), a cool-season grass commonly found in the U.S. northern Great Plains. This provides a unique opportunity for cattle grazing systems to improve nutrient retention and recycling, resulting in a more sustainable grassland that benefits the forage and improves weight gains and economic returns for cattle producers. Therefore, a four-year study was conducted to examine the effects of dried distillers grains supplementation and fertilization strategies on cattle performance, forage growth characteristics, N and P recovery, estimated greenhouse gas emissions, and economics of stocker cattle grazing Plains Old World bluestem.

## 2. Materials and Methods

### 2.1. Research Site

Four grazing trials were conducted in the summers of 2010 to 2013 at the Crosstimbers-Bluestem Stocker Range (36°04′05″ N, 97°11′54″ W), located 11 km southwest of Stillwater, OK, USA. The primary soil types at this site are Coyle loam, Coyle–Lucien complex, Grainola–Lucien complex, Renfrow loam, Stephenville–Damell complex, Stephenville fine sandy loam, and Zaneis loam. Twelve pastures of Plains Old World bluestem (*Bothriochloa ischaemum* L. Keng. var *ischaemum*), ranging in size from 4.1 to 10.6 hectares (averaging 8.7 hectares), were used in this study. The pastures were seeded and established at this site in 1989.

### 2.2. Experimental Design and Treatments

Pastures were blocked by 1 of 3 locations and randomly assigned to one of four treatments: (1) low input, targeted stocking rate of 330 kg of BW/ha, and no fertilizer or distillers grains supplementation (LOW); (2) targeted stocking rate of 660 kg of BW/ha and no fertilizer, with distillers grains supplementation at a level of 0.75% of BW/day as-fed basis (DDGS), prorated for a 5-d per week feeding program; (3) targeted stocking rate of 660 kg of BW/ha and 90 kg of N/ha with no distillers grains supplementation (NFERT); and (4) targeted stocking rate of 660 kg of BW/ha, 90 kg of N/ha, and 39 kg of P/ha with no distillers grains supplementation (NPFERT). Nitrogen fertilizer application rates were based on previous data, and the phosphorus application rate was based on an initial soil test. The NFERT treatment is considered to be the typical production system for this region and forage species. Following the mid-point of the trial in each year, all cattle not in the DDGS treatment were supplemented with a 0.45 kg/day as-fed basis of a 40 % CP cottonseed meal-based supplement containing monensin (Table 1), prorated for 3 d per week feeding program. Pastures were maintained on the same treatments for all four years. Cattle were stratified by initial BW and randomly assigned to 1 of 12 pastures. Fertilizer was applied as urea and diammonium phosphate to pastures in single applications on 29 April 2010; 4 May 2011; 2 May 2012; and 16–18 May 2013. Broadleaf herbicide was also applied to all pastures on 29 April 2010. All cattle had ad libitum access to rural water in open tanks and plain salt throughout the grazing season.

### 2.3. Cattle and Measurements

In 2010, 2011, 2012, and 2013, 224 heifers (274 ± 33 kg BW), 233 steers (237.9 ± 23 kg), 230 heifers (266.2 ± 28.1 kg), and 268 steer calves (219.4 ± 37.0 kg), respectively, purchased by a third party, were used in the trials. In each year, cattle were randomly assigned to 1 of 12 pastures to achieve the desired stocking density. Trials began on 18 May 2010; 17 May 2011; 17 May 2012; and 21 May 2013, and lasted for 133, 63, 119, and 121 d in 2010, 2011, 2012, and 2013, respectively. Cattle were weighed at the start, mid-point, and end of the trial after 12 h of removal from feed and water to minimize the effect of gut fill, except for 2011, where the trial was terminated on day 63 because of inadequate amounts of forage and the possibility of forage stand damage from continued grazing.

### 2.4. Cattle Management

In 2010, 2011, and 2012, cattle were administered a modified-live virus respiratory vaccine (Bovi-Shield Gold, Pfizer Animal Health; Florham Park, NJ or Titanium 3, AgriLabs, St. Joseph, MO, USA), 7-way clostridial vaccine (Vision 7 with Spur, Merck Animal Health; Summit, NJ), injectable dewormer (Dectomax Injectable, Zoetis, Florham Park, NJ, USA), and estradiol–trenbolone acetate combination implant (Component TE-G with Tylan, Elanco Animal Health; Greenfield, IN, USA) upon arrival at the Crosstimbers Bluestem Stocker Range. In 2013, the cattle were vaccinated as suckling calves with a preventative killed virus respiratory vaccine (Triangle 9 + PH-k, Boehringer Ingelheim Pharmaceuticals Inc., Ridgefield, CT, USA), a clostridial vaccine (Covexin 8, Merck Animal Health, Millsboro, DE, USA), a modified live virus respiratory vaccine (Vista Once SQ, Intervet, Millsboro, DE, USA), and a zeranol anabolic implant (Ralgro, Merck Animal Health, Millsboro, DE, USA) before arrival at the Crosstimbers Bluestem Stocker Range. Steers were revaccinated with Vista Once SQ at the time of trial initiation. At the mid-point and continuing throughout the remainder of each trial, all cattle not receiving the DDGS treatment were provided the monensin-containing protein supplement. This was carried out to maintain acceptable gains throughout the rest of the growing season, as forage CP decreased with plant maturity.

### 2.5. Forage Sampling Procedures

In each year of the study, standing forage biomass and diet quality samples were collected once per month throughout the trial. Biomass samples were collected at approximately 1 sample per 2 hectares per pasture, using GPS units to ensure that the samples were collected from approximately the same locations within each pasture at each time point across the years. Biomass samples were collected using a 0.19 m^2^ frame as a clipping guide, and the forage was hand-clipped to ground level. End-of-season forage yield samples were also collected at the end of the growing season in 2010 and 2011 from 3 grazing exclosures (approximately 1.67 m^2^ each using panels 15 m tall) per pasture. One sample per exclosure was collected using a 0.19 m^2^ frame. Three samples from three locations in each pasture (approximate front 1/3, middle 1/3, and back 1/3) were collected monthly for the measurement of forage nutritive value by hand-clipping the top 1/3 of the standing forage in several sub-locations. Samples of distillers grains and protein supplements were collected weekly throughout the grazing trials and were composited by month for later analysis. Forage samples were ground and composited by pasture within clipping date.

### 2.6. Laboratory Analysis

All forage, and distillers grains and protein supplement samples were dried at 55 °C in forced-air ovens to constant weights. Dried weights were used to calculate forage and feed DM content and the standing forage biomass (kg DM/hectare). Forage diet quality and feed supplement samples were then ground through a 2 mm screen in a Wiley mill (Thomas Scientific, Philadelphia, PA, USA). Forage quality samples were analyzed for ash (combusted in a 500 °C muffle furnace), CP (%N × 6.25; Truspec-CN LECO Corporation, St. Joseph, MI, USA), and sequential NDF-ADF (Ankom Tech Corporation, Fairport, NY, USA) at the Oklahoma State University Ruminant Nutrition Laboratory. Feed samples were analyzed for CP, NDF, and ADF at the Dairy One Forage Laboratory (Ithaca, NY, USA). Total digestible nutrient (TDN) values for distillers grains and protein supplement samples were taken from Dairy One Forage Laboratory reports, and the values for forage were calculated using the following equation: TDN, % = 88.9 – (0.779 × ADF), where ADF is expressed as a percentage of dry matter. Mineral analysis of the forage, distillers grains, and protein supplements was conducted by the Oklahoma State University Soil, Water, and Forage Analytical Laboratory.

### 2.7. Economic Analysis

Historical prices were collected from the USDA Agricultural Marketing Service (AMS; https://mymarketnews.ams.usda.gov) and the Economic Research Service (ERS; https://data.ers.usda.gov/FEED-GRAINS-custom-query.aspx). The prices used for dried distillers grains, protein supplement ingredients (cottonseed meal, soybean meal, and wheat middlings), and urea and diammonium phosphate fertilizer were based on the first week of April each year using regional values from Kansas City for feedstuffs and national values for fertilizer. The purchase price of cattle was based on market reports for the first week of April from Joplin Regional Stockyards (Carthage, MO, USA). The average initial body weight of the cattle in each year was used to determine the purchase price, assuming that the cattle for each treatment were bought together. The sale prices of cattle were based on market reports for the last week of September (except 2011) from Oklahoma National Stockyards (Oklahoma City, OK, USA). The average final body weight of the cattle in each pasture was used to determine the sale price using a price slide developed using prices for a 90 kg range, around the mean final body weight of cattle in all pastures. Variable expenses were computed based on input prices and the amount of feed and fertilizer used during the trial on a per-head and per-hectare basis. Even though the DDGS cattle had to be fed daily, regardless of the system, cattle needed to be checked for health issues daily; thus, labor was not included as it was expected to be similar among the treatments. The fixed costs of land and facilities were considered to be the same among the treatments. Revenue was computed as the sale price multiplied by the final body weight, and returns were computed as revenue minus input costs and cattle purchase cost on a per head and per hectare basis. These were considered returns to land, labor, and management.

Additionally, a sensitivity analysis was performed comparing DDGS and NFERT management systems. The average cattle performance and prices, feed consumption and prices, and fertilizer use and prices were used. For the DDGS system, the price of dried distillers grains was adjusted by ±5%, ±10%, and ±15% from the average price. For the NFERT system, the price of urea fertilizer was adjusted by ±5%, ±10%, and ±15% from the average price, and the average price of protein supplements was kept constant. The difference in returns per head of DDGS minus NFERT was computed such that a positive value indicated that DDGS would be more profitable, and a negative value indicated that NFERT would be more profitable.

### 2.8. Nutrient Retention by Cattle

Nitrogen inputs for each pasture included the feed N consumed, as well as the fertilizer N applied and an estimate of atmospheric N deposition from the National Atmospheric Deposition Program’s Oklahoma collection sites (https://nadp.slh.wisc.edu/networks/national-trends-network/). Nitrogen recovery was calculated as N retained in BW gain, divided by total N inputs and reported as a percentage of recovery. The protein content of the BW gain of the cattle was calculated from equations of the National Academy of Science, Engineering, and Medicine (NASEM), equation numbers from reference [10].

Eq. 12-5; EBG, kg = 0.956 × SWG(1)

Eq. 12-9; EQSBW, kg = SBW × (SRW/FSBW) (2)

Eq. 12-4; EQEBW, kg = 0.891 × EQSBW (3)

Eq. 12-1; RE, Mcal/day = 0.0635 × EQEBW^0.75^ × EBG^1.097^ (4)

Eq. 12-8; NPg, g/day = SWG × (268 − (29.4(RE/SWG)))(5)

NN, g/day = NP × 0.16 (6)

where EBG is empty body gain; SWG is shrunk weight gain in kg; EQSBW is equivalent shrunk body weight; SBW is average shrunk body weight during the trial in kg; SRW is the standard reference weight for the expected final body fat (478 kg for animals finishing at small marbling or 28 % body fat); FSBW is the estimated final shrunk body weight at the body fat endpoint (542, 558, 593, and 567 kg were used in 2010, 2011, 2012, and 2013, respectively, based on USDA Agricultural Marketing Service Direct Slaughter Cattle reports for the months and years in which cattle would be expected to finish); EQEBW is equivalent empty body weight; RE is retained energy in Mcal/day; NPg is net protein gain in g/day; and NN is net nitrogen gain in g/day.

Phosphorus inputs for each pasture included feed P consumed and fertilizer P applied. Phosphorus recovery was calculated as P retained in BW gain divided by total P inputs and reported as a percentage of recovery. The phosphorus content of BW gain was calculated from equations in NRC, Table 10-2 from reference [11].

Table 10-2; NP, g/day = NPg × 0.045 (7)

where NP is the net phosphorus gain and NPg is the net protein gain in g/day.

### 2.9. Greenhouse Gas Emissions

#### 2.9.1. Enteric Methane

Enteric methane emissions were calculated based on diet digestibility and equations of the Intergovernmental Panel on Climate Change (IPCC) [7]. Diet digestibility was calculated from the combination of forage and supplement intake and TDN values. Forage DMI was estimated using an iterative process in Microsoft Excel from the observed shrunk weight gain; estimated final shrunk BW at harvest; observed supplement intake and TDN value; observed forage TDN value; dietary energy conversion equations of NASEM [10]; and growth equations of NASEM [10], outlined above. Methane emissions were then calculated using the following equations from IPCC, equation numbers from reference [7].

Eq. 10.14; REM = [1.123 − (4.092 × 10^−3^ × DE) + (1.126 × 10^−5^ × bDE^2^) − (25.4/DE)] (8)

Eq. 10.15; REG = [1.164 − (5.160 × 10^−3^ × DE) + (1.308 × 10^−5^ × DE^2^) − (37.4/DE)] (9)

Eq. 10.16; GE, MJ/head/day = [(Nem/REM) + (NEg/REG)]/DE(10)

Eq. 10.21; EF, kg CH_4_/head/day = [GE × (Ym/100)]/55.65 (11)

where REM is the ratio of diet net energy for maintenance to dietary digestible energy; DE is diet digestible energy in %; REG is the ratio of diet net energy for gain to diet digestible energy; GE is gross energy; NEm is net energy for maintenance in MJ/head/day; NEg is net energy for gain in MJ/head/day; EF is the emissions factor per animal; and Ym is the methane conversion factor (6.5%; in Table 10.12 from reference [7]). Methane emissions were converted to carbon dioxide equivalents using a factor of 25.

No attempt was made to adjust enteric methane emissions for the inclusion of monensin in the cottonseed meal-based supplement. Meta-analyses have indicated that monensin can reduce methane production in short-term studies lasting fewer than 80 days [12,13], but both studies suggest that long-term use of ionophores is limited due the transient nature of the effect. Sauer et al. [14] reported that dairy cows previously fed monensin where there was a reduction in methane production had undergone an adaptation such that subsequent monensin administration did not reduce methane production. However, Odongo et al. [15] reported continued reduction in methane production with monensin feeding for 6 months in dairy cows fed a 60% forage diet. Additionally, studies [16,17] in which beef cattle were fed high-forage diets (>60% forage) found no effect of monensin on methane yield (g/kg DMI). The IPCC [7] does not make adjustments to the methane conversion factor for inclusion of monensin in the diet; thus, to be consistent with the IPCC [7] guidelines, no adjustment for monensin inclusion was made.

#### 2.9.2. Manure Methane

Methane emissions from excreted manure onto the pastures were computed using the following equations from IPCC, equation number from reference [7].

Eq. 10.24; VS, kg/head/day = [GE × (1 − DE/100) + (UE × GE)] × (1 − ASH/18.45)(12)

Table 10.17; EF, kg CH_4_/head/day = (VS × B_o_ × MCF/100 × 0.67) (13)

where VS is volatile solids in manure; UE is the urinary energy as fraction of GE, with a default value of 0.04 used; ASH is the ash content of manure as a fraction of DMI, where a value of 0.10 was used based on diet ash concentration and diet DM digestibility; B_o_ is the maximum methane-producing capacity of manure (0.19; in Table 10A-5 from reference [7]); and MCF is the methane conversion factor, representing the degree to which B_o_ is achieved (1.0%; in Table 10.17 from reference [7]). Methane emissions were converted to carbon dioxide equivalents using a factor of 25.

#### 2.9.3. Nitrous Oxide

Nitrous oxide emissions from excreted manure onto the pastures were calculated according to the nitrogen excreted based on nitrogen intake and the nitrogen retained in BW gain using equations from IPCC [7].

Eq. 10.25; N_2_O, kg/head/day = Nex × EF × 44/28 (14)

where Nex is the amount of nitrogen excreted in kg/head/d and EF is the emissions factor for direct N_2_O loss from manure nitrogen (0.02; in Table 11.1 from reference [7]). Nitrous oxide emissions from nitrogen fertilizer application to the pastures were calculated using the equation from IPCC [7].

Eq. 11.2; N_2_O, kg/ha = Napp × EF × 44/28 (15)

where Napp is the amount of N fertilizer applied in Mg/ha and EF is the emissions factor for N_2_O loss from inorganic fertilizer (0.01; in Table 11.1 from reference [7]). Nitrous oxide emissions were converted to carbon dioxide equivalents using a factor of 298.

#### 2.9.4. Indirect Emissions

Carbon dioxide equivalent emissions for fertilizer manufacturing were calculated using data obtained from West and Marland [18]. The carbon dioxide equivalent emissions for the production of dried distillers grains plus solubles were calculated based on efficiency of the ethanol yield [19] and the life cycle assessment for corn-based ethanol by Kim and Dale [20]. Carbon dioxide equivalent emissions for the production of the 40% protein supplement were based on soybean meal yield [21] and life cycle assessment for soybean oil production [20]. Data for soybean meal were used in place of cottonseed meal for two reasons: (1) a comprehensive life cycle assessment of cottonseed meal/oil production in the U.S. is not available, and (2) replacement of cottonseed meal with soybean meal in the protein supplement would result in similar animal performance at the same supplementation rate.

### 2.10. Nitrous Oxide Flux

Nitrous oxide flux measurements were made following nitrogen fertilizer application rates in small plots in Plains Old World Bluestem pastures in 2010. Fertilizer was applied as urea on 10 May at 3 application rates of 0, 67.5, 101, and 135 kg N/ha, and this was repeated 3 times. Nitrous oxide emissions were measured using static chambers on days 0, 2, 7, 14, 22, 29, 36, 43, 50, 58, 64, 71, 77, 91, and 106 after application. Static chambers measuring 38.1 by 12.7 cm, designed in accordance with the USDA-ARS GRACEnet Project Protocols [22], were installed in each plot. On each sampling day, 20 mL gas samples were withdrawn at 0, 15, and 30 minutes from the deployed chambers and injected into 30ml glass vials sealed with butyl rubber septum. Gas samples were analyzed using a Varian 450-GC (Agilent, Santa Clara, CA, USA) with an electron capture detector. Nitrous oxide fluxes were calculated from the linear increase in gas concentrations in the chamber headspace versus time, as described by Parkin and Venterea [22].

### 2.11. Statistical Analysis

Cattle performance, end-of-season forage yield, N and P recovery, and economic data were analyzed with a randomized complete block design using the *lmer* function of the *lme4* package for R statistical software (version 4.0.4; https://www.rdocumentation.org/ (accessed on 1 December 2022)). Pasture was the experimental unit and block was considered as a random effect, with treatment, year, and treatment × year interaction as fixed effects. The model for analysis of standing forage biomass and nutritive value included fixed effects of treatment, year, sampling day within year, treatment × year interaction, and treatment × sampling day within year interaction, as well as a random effect of block. Data were analyzed using the *lmer* function of the *lme4* package. The sampling day differed between years and, thus, was modeled as a continuous variable in an analysis of covariance (ANCOVA) rather than a repeated measure. Year was considered a fixed effect due to widely different weather and forage production between years, and the interest in testing whether the treatment effects were similar among different years. All models used an unstructured correlation for the variance–covariance matrix based on Aikaike’s Information Criteria (AIC), which used the *AIC* function in the base stats package. The *anova* and *summary* functions of the base stats package were used to evaluate the significance. Least square means were computed using the *emmeans* function in the *emmeans* package by means of Tukey’s W adjusted pairwise comparisons, with significance set at *p* ≤ 0.05. Nitrous oxide flux was analyzed as cumulative emissions over the 106-day measurement period using a linear regression mixed model with the *lmer* function. The nitrogen fertilizer application rate was modeled as a linear and quadratic predictor of cumulative N_2_O emissions with random intercept and slope for replicate. Predictors were evaluated using the *anova* function and considered to be significant at *p* ≤ 0.05.

## 3. Results

**Figure 1:**
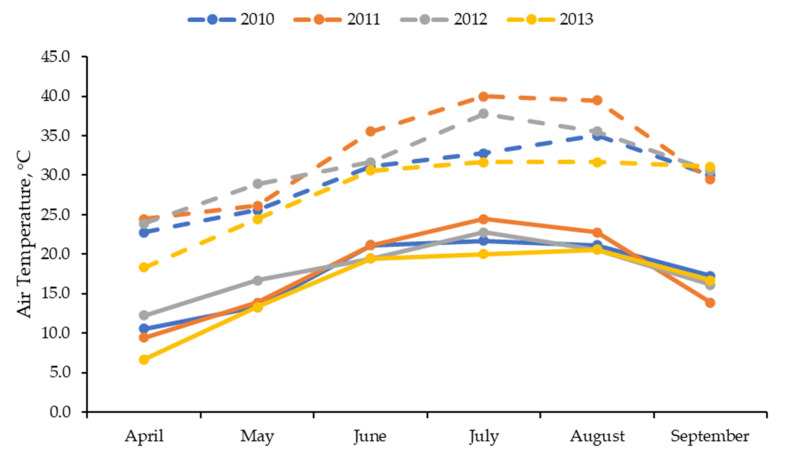
Average monthly maximum (dash lines) and minimum (solid lines) daily temperature during the grazing season in each of the 4 years.

**Figure 2:**
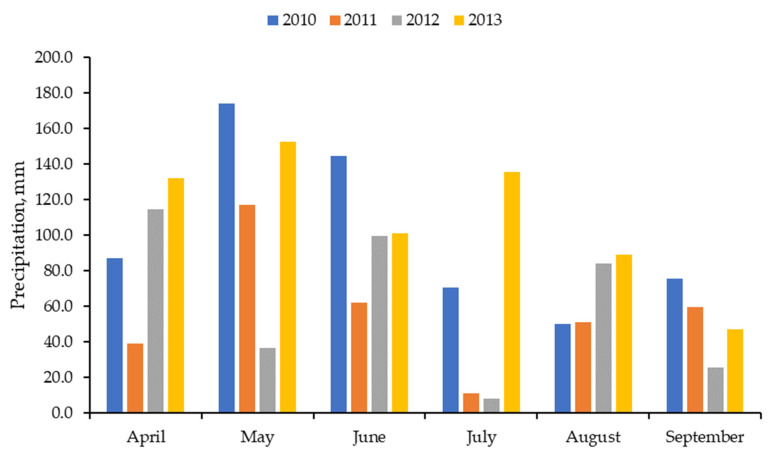
Total monthly precipitation during the grazing season in each of the 4 years.

Because of variations in cattle weights during each year, the actual stocking rates varied (LOW = 355, 389, 333, and 302 kg/ha; DDGS 695, 706, 651, and 614 kg/ha; NFERT = 659, 716, 632, and 610 kg/ha; NPFERT 683, 718, 655, and 622 kg/ha for 2010, 2011, 2012, and 2013, respectively). This resulted in DDGS, NFERT, and NPFERT having stocking rates 1.91 times greater than LOW on average, which was intended. The maximum ambient temperature was warmer for June, July, and August in 2011 than the other 3 years, whereas minimum temperatures were more similar among years (Figure 1). Precipitation patterns differed among years, with 2012 having a much drier May and 2011 and 2012 having a much drier July than other years (Figure 2). Additionally, the total precipitation from April through September was much less in 2011 and 2012 than in 2010 and 2013 (340 and 367 vs. 601 and 656 mm, respectively).

**Figure 3:**
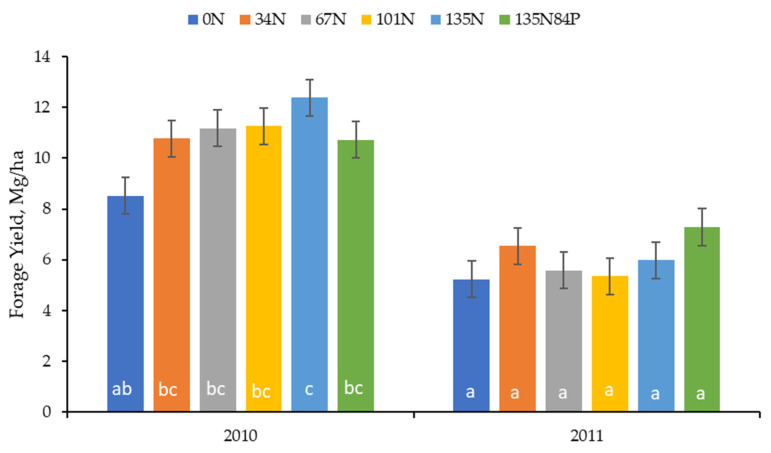
Forage yield from nitrogen and phosphorus application in Plains Old World Bluestem plots at rates of 0 (0N), 34 (34N), 67 (67N), 101 (101N), and 135 (135N) kg N/ha, and 135 kg N/ha + 84 kg P/ha (135N84P). Treatment: *p* = 0.01; Year: *P* = 0.01; Treatment*Year: *p* = 0.02. ^abc^ Bars without a common letter within year are different at *p* ≤ 0.05.

The forage yield from the nitrogen fertilizer rate evaluation was greater at 135 kg N/ha than 0 kg N/ha, with 34, 67, and 101 kg N/ha being intermediate in 2010, indicating that the forage yield generally increased linearly with nitrogen application (Figure 3). Phosphorus application did not affect the forage yield. There was no difference among nitrogen application rates in 2011, most likely due to the lack of precipitation in 2011 (Figure 2).

**Figure 4:**
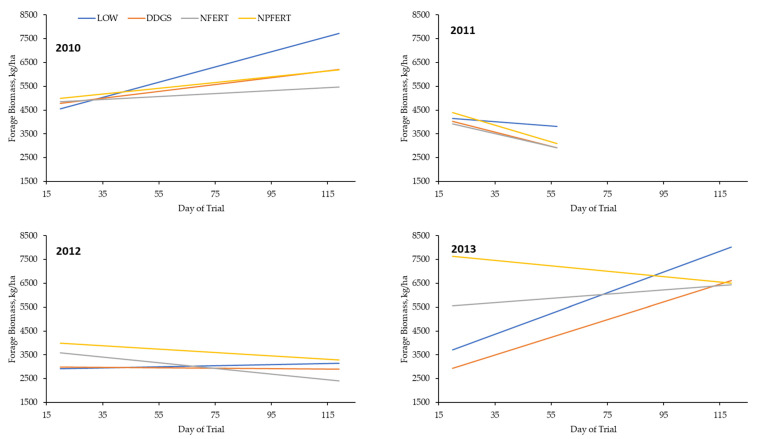
Forage biomass among treatments according to day of trial within a year. LOW = targeted stocking rate of 330 kg of BW/ha and no fertilizer or DDGS supplementation; DDGS = targeted stocking rate of 660 kg of BW/ha and no fertilizer with DDGS supplementation at a level of 0.75% of BW/day; NFERT = targeted stocking rate of 660 kg of BW/ha and 90 kg of N/ha with no DDGS supplementation; NPFERT = targeted stocking rate of 660 kg of BW/ha, 90 kg of N/ha, and 39 kg of P/ha with no DDGS supplementation. Treatment: *p* = 0.01; Year: *p* = 0.07; Treatment*Year: *p* = 0.04; Day(Year): *p* = 0.01; Treatment*Day(Year): *p* = 0.01.

**Figure 5:**
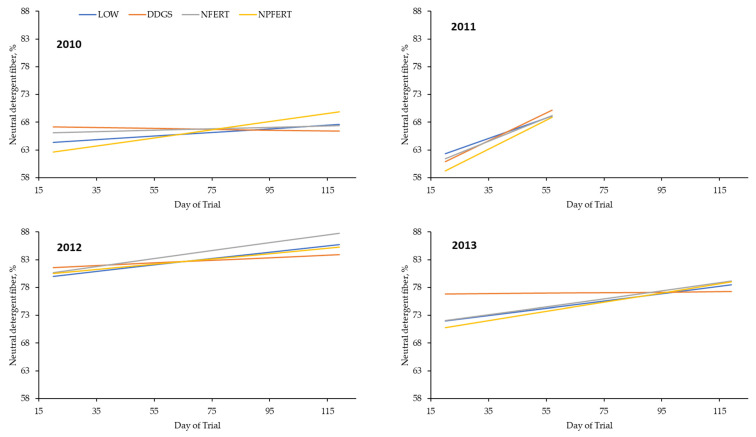
Forage neutral detergent fiber (NDF) concentration among treatments according to day of trial within a year. LOW = targeted stocking rate of 330 kg of BW/ha and no fertilizer or DDGS supplementation; DDGS = targeted stocking rate of 660 kg of BW/ha and no fertilizer with DDGS supplementation at a level of 0.75% of BW/day; NFERT = targeted stocking rate of 660 kg of BW/ha, and 90 kg of N/ha with no DDGS supplementation; NPFERT = targeted stocking rate of 660 kg of BW/ha, 90 kg of N/ha, and 39 kg of P/ha with no DDGS supplementation. Treatment: *p* = 0.07; Year: *p* = 0.01; Treatment*Year: *p* = 0.36; Day(Year): *p* = 0.01; Treatment*Day(Year): *p* = 0.04.

The standing forage biomass was affected (*p* ≤ 0.05) by a treatment × year interaction, where DDGS had a lower (*p* ≤ 0.05) biomass than NPFERT, with LOW and NFERT being intermediate in 2013 and no difference (*p* > 0.05) among treatments in the other years (Table 2). There was also a treatment × day (year) interaction for standing forage biomass, where LOW and DDGS had greater (*p* ≤ 0.05) slopes across the days than NPFERT, with NFERT being intermediate in 2013 and no difference (*p* > 0.05) among treatments in other years (Table 3; Figure 4). The forage CP was greater (*p* ≤ 0.05) for NPFERT than LOW, with DDGS and NFERT being intermediate, and forage CP decreased (*p* ≤ 0.05) as the days of the trial progressed. Forage CP was different (*p* ≤ 0.05) among all years, with a ranking from least to greatest of 2011, 2010, 2012, and 2013. Forage P was greater (*p* ≤ 0.05) for NPFERT than LOW and NFERT, which were not different (*p* > 0.05), with DDGS being intermediate. In 2011, the forage P was lower (*p* ≤ 0.05) than in other years, 2010 and 2011 were not different (*p* > 0.05), and the forage P was greater (*p* ≤ 0.05) in 2012 than in other years. The phosphorus concentration of the forage decreased (*p* ≤ 0.05) as the days of the trial progressed. Forage NDF differed (*p* ≤ 0.05) among all years, with a ranking from least to greatest of 2010, 2011, 2013, and 2012. Even though the F-test for the effects of treatment and treatment × day(year) were significant (*p* ≤ 0.05), there were no differences (*p* > 0.05) in the forage NDF among pairwise comparisons of treatments (Figure 5). Treatment did not influence (*p* > 0.05) forage ADF or TDN, but forage ADF was greater (*p* ≤ 0.05) and TDN lesser (*p* ≤ 0.05) in 2012 than 2013, which was a different result (*p* ≤ 0.05) compared to 2010 and 2011, which were not different (*p* > 0.05). Forage ADF increased (*p* ≤ 0.05) and TDN decreased (*p* ≤ 0.05) as the days of the trial progressed. The end-of-season forage yield was lesser (*p* ≤ 0.05) for DDGS than NPFERT, with LOW and NFERT being intermediate, and was greater (*p* ≤ 0.05) in 2010 than in 2011 (Table 4).

The initial BW differed (*p* ≤ 0.05) among years, with 2011 and 2013 having lower BW values than 2010 and 2012, but the initial BW values were similar (*p* > 0.05) among treatments (Table 4). Consequently, the final BW values were also lesser (*p* ≤ 0.05) in 2011 and 2013 than in 2010 and 2012, but DDGS had greater (*p* ≤ 0.05) final BWs than NFERT and NPFERT, with LOW being intermediate. Interestingly, there was a treatment × year interaction for ADG, where LOW and DDGS had greater (*p* ≤ 0.05) ADG values than NFERT and NPFERT in 2010, DDGS and NPFERT had greater (*p* ≤ 0.05) ADG values than LOW and NFERT in 2011, DDGS had greater (*p* ≤ 0.05) ADG values than all other treatments in 2012, and there was no difference (*p* > 0.05) in ADG among treatments in 2013. 

The gain per hectare followed a similar pattern to the stocking rate among treatments, with DDGS, NFERT, and NPFERT having greater (*p* ≤ 0.05) gains than LOW, but DDGS had a greater (*p* ≤ 0.05) gain than NFERT and NPFERT, likely due to additional energy intake from distillers grains supplementation. The gain per hectare was greater (*p* ≤ 0.05) in 2010 than 2013, and this value was greater (*p* ≤ 0.05) than in 2011 and 2012. The estimated forage DMI was lesser (*p* ≤ 0.05) for DDGS than for other treatments, with LOW, NFERT, and NPFERT being not different (*p* > 0.05) in each year, even though there was a significant (*p* ≤ 0.05) treatment × year interaction. Supplement intake over the grazing trial followed a similar pattern as forage DMI, with DDGS being greater than other treatments in each year.

There was a treatment × year interaction for feed, fertilizer, and total input costs, primarily due to the lack of input costs for some treatments in 2011 (Table 5). Feed costs were greater (*p* ≤ 0.05) for DDGS than other treatments, and fertilizer costs were greater (*p* ≤ 0.05) for NFERT and NPFERT than other treatments in each year. The total input costs were lowest (*p* ≤ 0.05) for LOW and greatest (*p* ≤ 0.05) for NPFERT each year, and NFERT had greater (*p* ≤ 0.05) input costs than DDGS in 2010, but not (*p* > 0.05) in other years. The revenue increased (*p* ≤ 0.05) as the years progressed (2010<2011<2012<2013) and was greater (*p* ≤ 0.05) for DDGS than NFERT, with LOW and NPFERT being intermediate. 

The returns per head were greater (*p* ≤ 0.05) in 2010 and 2013, which were not different (*p* > 0.05), than 2012, which was greater (*p* ≤ 0.05) than 2011. Additionally, the returns per head were greater (*p* ≤ 0.05) for LOW and DDGS, which were not different (*p* > 0.05), than NFERT and NPFERT, which were also not different (*p* > 0.05). There was a treatment × year interaction for returns per hectare, where LOW had lower (*p* ≤ 0.05) returns than other treatments in 2010 and 2013, was not different (*p* > 0.05) from other treatments in 2011, and was only lower (*p* ≤ 0.05) than DDGS in 2012. The returns per hectare were not different (*p* > 0.05) between DDGS and NFERT in any year, and NFERT and NPFERT were not different (*p* > 0.05) in any year, but DDGS had greater (*p* ≤ 0.05) returns than NPFERT in 2010 and 2012.

In the sensitivity analysis, there was no scenario where NFERT was more profitable than DDGS (Table 6). With a 30% reduction in urea fertilizer price (USD 426/Mg) and a 30% increase in dried distillers grains price (USD 280/Mg), the DDGS system is only marginally more profitable at USD 1.62/hd greater. But with a 30% increase in urea fertilizer price (USD 790/Mg) and a 30% reduction in dried distillers grains price (USD 151/Mg), the DDGS system was substantially more profitable at USD 65.52/hd. Re-evaluating the analysis using 2022 prices (USD 345/Mg for dried distillers grains and USD 563/Mg for urea fertilizer) yielded very different results. The NFERT system is more profitable than the DDGS system at the average price (0%), with USD 18.41 greater returns. The DDGS system is only more profitable than the NFERT system when the dried distillers grains price is lower than average and the urea fertilizer price is greater than average.

There was a treatment × year interaction for N inputs; however, NFERT and NPFERT had similar (*p* > 0.05) N inputs, which were greater (*p* ≤ 0.05) than DDGS and LOW, and DDGS had greater (*p* ≤ 0.05) N inputs than LOW in all years (Table 7). The retained N was greater (*p* ≤ 0.05) in 2010 and 2013 than in 2011 and 2012 and was greater (*p* ≤ 0.05) for DDGS than NFERT, with NPFERT being intermediate; all treatments had greater (*p* ≤ 0.05) retained N than LOW. Treatment and year interacted (*p* ≤ 0.05) to influence the nitrogen use efficiency (NUE). The nitrogen use efficiency was greatest (*p* ≤ 0.05) for LOW in all years, with DDGS having a greater (*p* ≤ 0.05) NUE than NFERT in 2010 and NFERT and NPFERT in 2011 and 2013, but not (*p* > 0.05) in 2012. The nitrogen use efficiency was not different (*p* > 0.05) between NFERT and NPFERT in any year.

Phosphorus inputs were affected (*p* ≤ 0.05) by a treatment × year interaction, with LOW and NFERT having lesser (*p* ≤ 0.05) P inputs than DDGS and NPFERT in all years; however, DDGS had greater (*p* ≤ 0.05) P inputs than NPFERT in all years except for 2011, which was likely due to the shorter grazing season that year. Retained P was greater (*p* ≤ 0.05) for DDGS than NFERT, with NPFERT being intermediate and all other treatments being greater (*p* ≤ 0.05) than LOW. The retained P was greater (*p* ≤ 0.05) in 2010 and 2013 than in 2011 and 2012. Phosphorus use efficiency (PUE) was greater (*p* ≤ 0.05) for LOW and NFERT, which were not different (*p* > 0.05), than for DDGS and NPFERT, which were not different (*p* > 0.05).

Estimated enteric and manure methane emissions were lesser for LOW than for other treatments, which is likely a function of the stocking rate (Table 8). The estimated enteric methane emissions were greater (*p* ≤ 0.05) in 2010 than 2012, with 2013 being intermediate, and enteric methane emissions in 2011 were lesser (*p* ≤ 0.05) than other years due to the shorter grazing season. The estimated manure methane emissions were lower (*p* ≤ 0.05) in 2011 than in other years. 

**Figure 6:**
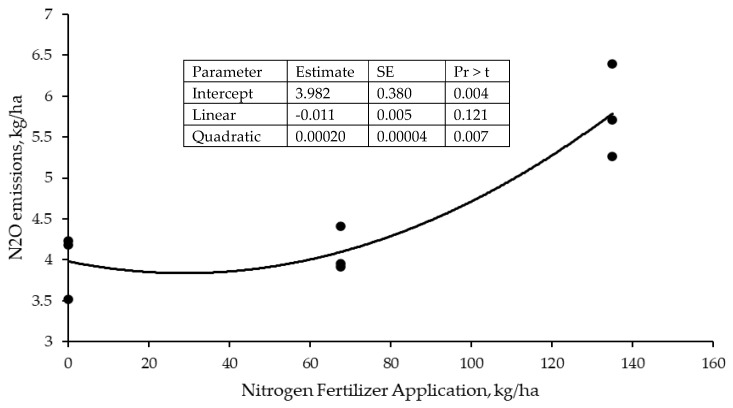
Relationship between nitrogen fertilizer application rate and nitrous oxide (N_2_O) emissions in Old World Bluestem pastures in 2010. SE = standard error; Pr > t = probability greater than t statistic. Root mean square error = 0.1658; R^2^ = 0.9428.

There was a treatment × year interaction (*p* ≤ 0.05) for estimated nitrous oxide emissions from manure, where LOW showed lower (*p* ≤ 0.05) emissions than DDGS, NFERT, and NPFERT, which were not different (*p* > 0.05), in 2010, 2012, and 2013, but the treatments were not different (*p* > 0.05) in 2011. Estimated nitrous oxide emissions from inorganic fertilizer application were greater (*p* ≤ 0.05) for NFERT and NPFERT, which were not different (*p* > 0.05), than LOW and DDGS, which were also not different (*p* > 0.05). In 2010, the observed nitrous oxide emissions from inorganic fertilizer increased at an increasing rate with an increasing nitrogen application rate (Figure 6). 

The total direct carbon dioxide equivalent emissions were lower (*p* ≤ 0.05) for LOW than other treatments, which were not different from each other (*p* > 0.05), most likely due to the lower stocking rate. They were also lower (*p* ≤ 0.05) in 2011 than in other years, which were not different (*p* > 0.05), most likely due to the shorter grazing season. The indirect carbon dioxide equivalent emissions were lesser (*p* ≤ 0.05) for LOW than DDGS, which was less (*p* ≤ 0.05) than NFERT and NPFERT, which were not different (*p* > 0.05). Total carbon dioxide equivalent emissions followed a similar pattern as direct emissions, where emissions were lesser (*p* ≤ 0.05) for LOW than other treatments, which were not different (*p* > 0.05), and were lesser (*p* ≤ 0.05) in 2011 than other years, which were not different (*p* > 0.05), likely due to the greater magnitude of direct than indirect emissions. However, when adjusted for weight gain per hectare, the total carbon dioxide equivalent emissions were not different (*p* > 0.05) between LOW and DDGS, and both were lesser (*p* ≤ 0.05) than NFERT and NPFERT, which were not different (*p* > 0.05) from each other. Additionally, the total carbon dioxide emissions per kilogram of weight gain were different (*p* ≤ 0.05) among all years, with a ranking from least to greatest of 2011, 2010, 2013, and 2012.

## 4. Discussion

### 4.1. Effect of Treatment × Year Interaction

The reason that the year was modeled as a fixed effect was due to the differences in weather and forage production among years. The 30-year average precipitation for April through September for this area is approximately 600 mm, indicating that 2010 was an average year, 2011 and 2012 were well below average, and 2013 was an above-average year. Differences between treatments were impacted by the effect of the year. 

One concern involved in replacing N fertilizer with feed supplement is the potential detrimental impact on forage production and stand persistence, which was not evident in the first three years of the study, but in 2013, after the two-year drought, the rebound in forage biomass was improved with nitrogen and phosphorus fertilization compared to feeding distillers grains, suggesting a lack of available nitrogen and phosphorus for plant growth in the DDGS system. In low-input grazing systems, grazing management plays an important role in the spatial distribution of excreted nutrients [23,24,25,26,27]. When replacing fertilizer application with distillers grains, excreted N and P from the feed supplement consumed by cattle is necessary to maintain soil nutrient levels for plant growth. In this study, continuous grazing management was used, but the results may have been different if management-intensive grazing had been used, as feces and urine are more evenly distributed across the pasture than in continuous grazing systems [27,28]. Additionally, maintaining a high stocking rate in the DDGS system likely improved nutrient recycling such that nutrients were more consistently available for plant growth [29].

In 2011, a drought and a lack of forage production necessitated the destocking of pastures at the mid-point of the trial, which altered the cattle ADG among treatments compared with the other years. Cattle in the systems which were provided with P in the form of distillers grains or fertilizer had greater gains, likely due to the lack of phosphorus availability in the soil during the drought [30,31]. Phosphorus fertilization during a drought may improve P uptake by overcoming low P diffusion in water-restricted environments [32]. In fact, the forage P concentration results illustrate the lack of P in the diet for LOW and NFERT, with an estimated P intake of 7, 27, 6, and 12 g/d for LOW, DDGS, NFERT, and NPFERT, respectively, compared with an estimated P requirement of 19 g/d [10]. In 2013, after the two-year drought, there was no difference in ADG among treatments, potentially due to the lower standing forage biomass when feeding distillers grains and high forage CP in that year.

In 2012, the second year of the drought, replacing the fertilizer with distillers grains supplements did not improve NUE, as in other years. In 2012, forage had the greatest NDF and ADF concentrations, which would have been expected to enhance the benefit of feeding distillers grains. The reason for the lack of response is unknown.

Nitrous oxide emissions from manure, which is a function of N excretion, were less for LOW than for the other treatments, except in 2011. Nitrogen excretion was lower in 2011 than other years due to the shorter grazing season, but the numerical ranking of treatments was similar to other years. The lack of difference may be due to the smaller magnitude of emissions because of the shorter grazing season and lesser total N excretion in 2011, relative to the standard error, than in other years.

### 4.2. Effect of Treatment

Similarly to our results, Greenquist et al. [8] reported that a low-input system for grazing smooth bromegrass had lesser forage CP than systems using N fertilizer, but unlike our results, standing forage biomass was greater for the system receiving N fertilizer than the low input system or distillers grains system. Greenquist et al. [8] used a different grazing management method than the current study, where cattle were rotationally grazed and grazing pressure was maintained by the use of put-and-take cattle. The rotational grazing management likely provided a greater forage rest period and, coupled with the N fertilizer, increased forage growth [33,34,35]. Treatment did not affect the estimated TDN of Old World bluestem in the current study, and similar treatments did not affect the in vitro dry matter digestibility of smooth bromegrass [8]. The lack of effect is likely due to the absence of differences in forage NDF and ADF, which are the primary determinants of forage digestibility [36,37,38].

Cattle fed distillers grains gained weight more quickly and were heavier at the end of grazing than those grazing fertilized pastures, and feeding with distillers grains increased the gain per hectare above other treatments without negatively impacting forage production throughout most of the trial. Additionally, feeding distillers grains maintained the crude protein and phosphorus concentration in forage compared with the fertilized pastures, whereas a lack of feed or fertilizer inputs did not. Likewise, replacing N fertilizer with distillers grains increased cattle gains in smooth bromegrass pastures, but did not maintain forage production or crude protein [8]. Old World bluestem has a greater response to N fertilization than bromegrass (45 vs. 33 kg forage per kg N) [39,40] and Old World bluestem converts inorganic N into plant protein more efficiently (22.7 vs. 27.2 kg N to increase CP by 1%) [39,40,41]; thus, the limited N input to the grazing system from distillers grains was able to maintain forage production and nutritive value in the Old World bluestem pastures of the current study more effectively than the smooth bromegrass pastures in Greenquist et al. [8]. Additionally, grazing cattle supplemented with distillers grains consumed, less forage [42] which agrees with the lesser estimated forage intake of cattle fed distillers grains and could have offset lesser forage production, resulting in similar standing forage biomass. In agreement with this hypothesis, forage yield was the lowest in the distillers grains system in 2010 and 2011.

Nitrogen and phosphorus use efficiency appear to be driven primarily by inputs. Stocker cattle grazing systems with the least inputs had the greatest efficiency, but also the lowest productivity, whereas those with the greatest productivity had the greatest inputs and lowest efficiency. Feeding distillers grains optimized this tradeoff, with high productivity and moderate inputs achieving moderate efficiency (current study, [9]). Increasing N fertilizer application rates increased the BW gain per hectare in Old World bluestem pastures, with a peak between 70 and 100 kg N/ha, but nitrogen use efficiency decreased quadratically with increasing N fertilizer [40], indicating the strong influence of N input on efficiency. There was also a tradeoff between maximizing nitrogen versus phosphorus use efficiency with productivity. The low-input system had the greatest NUE and PUE, but the least productivity, whereas replacing the fertilizer with distillers grains improved the productivity and NUE, but maintained or decreased PUE depending upon whether P fertilizer was included. The tradeoff between NUE and PUE when feeding distillers grains was likely due to the level of input; DDGS had moderate N input, but the greatest P input among treatments.

Increased stocking results in greater estimated greenhouse gas emissions [43], as was found with DDGS, NFERT, and NPFERT, but this is not always the case, at least with methane emissions [44], as both low and high stocking rates can result in the consumption of forage with lesser nutritive value [45,46,47,48,49]. Feed or forage intake is the primary driver of daily methane emissions [50,51,52,53], but diet digestibility impacts the intensity of methane emissions (g CH_4_/kg DMI) [50,51,54]. Additionally, total nitrous oxide emissions increased with nitrogen fertilization, as would be expected, but nitrous oxide emissions from inorganic nitrogen fertilizer application may have been greater than estimated. The IPCC [7] recommends using an emissions factor of 1% to estimate nitrous oxide emissions from inorganic nitrogen fertilizer application, but the observed nitrous oxide emissions at 90 kg N/ha were approximately 3% of nitrogen applied, which would increase the greenhouse gas emissions of NFERT and NPFERT.

Interestingly, feeding with distillers grains resulted in lower indirect carbon emissions than fertilization. More importantly, feeding distillers grains resulted in a total carbon emissions intensity (CO_2_ equivalents/kg BW gain) similar to that of the low-input system and lower than fertilizer application while increasing the gain per hectare. Alemu et al. [55] and Wang et al. [56] reported that higher stocking rates and weaning weight per hectare in a cow-calf grazing system decreased the carbon emissions’ intensity, where enteric methane emissions were estimated to account for the most emissions, as in the current study. Strategically using natural resources and optimizing the associated carbon emissions to produce highly nutritious feedstuffs such as distillers grains is likely to improve the carbon footprint of beef production. According to the FAO [57] statistics, countries with lower carbon emissions intensities due to beef production are associated with greater intensities involved in the grain finishing of cattle. Additionally, grain-finishing production systems consistently have lower carbon emissions intensities [58,59,60]. Greenhouse gas emissions associated with grain crop production increase feed energy per unit of gas emissions compared with forage crop production, thus reducing the carbon emissions intensity of beef production.

During the second half of the trial, except in 2011, the LOW, NFERT, and NPFERT were fed a protein supplement providing 120 mg/day of monensin, which is part of the typical production for stocker cattle in Old World Bluestem pastures. Monensin was not included in the distillers grains fed to DDGS. The inclusion of monensin in a protein supplement increases ADG by 10% in stocker cattle [61] and would be expected to increase nitrogen retention in a stocker cattle production system. Despite the benefits of monensin, the differences in ADG were not consistent between cattle that received monensin and those that did not at similar stocking rates. Additionally, cattle fed the monensin-containing protein supplement did not exhibit greater net returns or nitrogen use efficiency than cattle fed distillers grains without monensin, except for the low stocking, low-input system. Thus, even though monensin likely increased the ADG of cattle, the alternative production system with distillers grains was more economically and environmentally sustainable than when monensin was fed to the typical production system. If monensin had been included in the distillers grains fed to DDGS cattle, the benefits of DDGS would likely have been even greater.

### 4.3. Effect of Year

Drought negatively impacts forage production [62,63,64,65], with approximately a 20% reduction in DM yield with light and moderate drought compared with a 37% reduction with severe drought [66], which may be influenced by grazing management [65,67]. But drought has varying effects on forage’s nutritive value. In a meta-analysis, Liu et al. [66] reported 8% reduction in the crude protein of forage with drought, whereas Sheaffer et al. [62] and Grant et al. [68] reported greater crude protein and lesser NDF and ADF during drought. The crude protein of alfalfa was unaffected by water stress, but leaf/stem ratio and in vitro dry matter digestibility increased with severe water stress [69,70].

Gain per hectare followed the pattern of forage production among years, which is expected. In 2012, the second year of the drought, gain per hectare was similar during a full season of grazing as it was in a half season of grazing in 2011. The ability to graze for the full season in 2012, even though precipitation was below average for the growing season, was due to the adequate precipitation during the previous fall and winter compared with 2011 (366 vs. 151 mm for 2012 and 2011, respectively). Thus, N and P retention were reduced during drought years, but NUE and PUE were not, again pointing to the fact that inputs are the driving force behind nutrient use efficiency, possibly due to the large magnitude of inputs relative to outputs.

In the low-input system, NUE and PUE declined over the years, indicating that the low-input system may not be sustainable over the long term. Berg [71] reported that soil N accumulation in fertilized pastures of Old World bluestem was very low, at ~8 kg/ha, and along with ~5 kg N/ha removed in cattle weight gain, this indicates that 75% of N inputs (atmospheric, feed supplement, fertilizer) were in the form of plant biomass or lost from the system. The nitrogen fertilizer application rate (35 to 105 kg/ha) had little impact on N recovery in the forage biomass of Old World bluestem, with an overall average of ~35% [2]. Thus, grazing systems require continual N inputs in order to be productive. 

Due to the length of the grazing season, the estimated enteric and manure methane emissions and direct and total carbon dioxide equivalent emissions were reduced in 2011. However, unlike NUE and PUE, the total carbon dioxide equivalent emissions per kilogram of BW gain appeared to be influenced by both emissions and productivity. The low productivity (gain per hectare), but short grazing season (lower emissions), in 2011 resulted in the lowest carbon emissions intensity, whereas the low productivity and long grazing season in 2012 resulted in the greatest carbon emissions intensity. White and Capper [72] estimated a 12% reduction in carbon equivalent emissions intensity when increasing ADG by 15%. 

### 4.4. Nitrous Oxide Emissions

The observed nitrous oxide emissions increased quadratically with the N fertilizer application rate, a result similar to those of previous studies [73,74,75]. At 90 kg N/ha, which was the application rate used in the grazing trials, the cumulative emissions over the grazing season were approximately 4.5 kg N_2_O/ha or 2.9 kg N_2_O-N/ha, which is an emissions factor of 3.2%, much greater than the emissions factor of 1.0% recommended by IPCC [7]. Others [76,77] have also measured larger emissions factors than recommended by IPCC [7]. Emissions are greater in grazed grasslands due to the increased soil organic matter decomposition, releasing N, than in other agricultural soils (crop and hay fields) [76,77,78], and are affected by soil type and climatic conditions [74,78,79]. Although fertilizer plots were not grazed in 2010, the plot area had been grazed the previous year such that dung and plant litter were likely influencing N_2_O emissions.

## 5. Conclusions

Nutrient cycling in grasslands is an important aspect to sustain functioning and maintain highly productive grassland. Annual nitrogen fertilizer application is expensive, but has the potential for considerable nitrogen losses to the environment, affecting the air and water quality. Low-input grazing systems have less environmental impact, but also less productivity. However, bringing nutrients into stocker cattle grazing systems through feed rather than fertilizer may improve weight gain and nitrogen use efficiency, and mitigate greenhouse gas emissions while capitalizing on nutrient cycling to maintain forage production. The reduced enteric methane emissions with increased digestibility of the diet, as well as the improved carbon footprint of producing cattle nutrients from field crops compared with forage, are important aspects leading to a more sustainable stocker cattle production system. Future production systems should look to capture efficiencies in other sectors of agriculture that can be utilized in beef production.

Our analysis made several assumptions. We assumed that increased nitrogen and phosphorus retention indicated less nitrogen and phosphorus loss from the production system. We assumed that the emissions factors used in large-scale life cycle assessments apply to stocker cattle production systems using Plains Old World bluestem, which may not be true, as was observed with the nitrous oxide flux experiment. Future research should further evaluate nitrogen and phosphorus losses, as well as greenhouse gas emissions, through empirical measurements.

## Figures and Tables

**Table 1:** Ingredients and chemical composition of supplements fed to grazing stocker cattle during the 4 years.

Item	Distiller’s Grains	Protein
Ingredient, % as-fed		
Dried distiller’s grains plus solubles	100.00	-
Cottonseed meal	-	80.50
Soybean meal	-	11.85
Wheat middlings	-	7.50
Rumensin 80	-	0.15
Nutrient (mean ± SD)		
Dry matter, %	89.70 ± 0.46	91.10 ± 0.68
Crude protein, % DM	31.38 ± 1.58	44.93 ± 1.83
Neutral detergent fiber, % DM	34.60 ± 3.34	26.40 ± 2.68
Acid detergent fiber, % DM	16.03 ± 2.04	14.85 ± 1.36
Calcium, % DM	0.04 ± 0.01	0.30 ± 0.02
Phosphorus, % DM	1.02 ± 0.08	1.20 ± 0.03
Magnesium, % DM	0.38 ± 0.03	0.67 ± 0.01
Sulfur, % DM	0.67 ± 0.15	0.48 ± 0.03
Potassium, % DM	1.28 ± 0.11	1.86 ± 0.02
Sodium, % DM	0.26 ± 0.09	0.17 ± 0.04
Total digestible nutrients ^1^, % DM	81.25 ± 1.92	78.75 ± 0.83

^1^ Total digestible nutrient values were computed by Dairy One Forage Laboratory (Ithaca, NY, USA).

**Table 2:** Forage biomass and nutritive value among treatments across 4 years in stocker steers and heifers.

Year	TRT ^1^	Biomass (kg/ha)	CP (%)	P (%)	NDF (%)	ADF (%)	TDN (%)
2010	LOW ^2^	6290 ^ab^	9.25 ^zZ^	0.15 ^yY^	65.87 ^Z^	33.67 ^X^	62.67 ^Z^
	DDGS	5562 ^b^	9.41 ^yzZ^	0.15 ^yzY^	66.83 ^Z^	34.34 ^X^	62.15 ^Z^
	NFERT	5192 ^bc^	10.23 ^yzZ^	0.15 ^yY^	66.68 ^Z^	33.14 ^X^	63.08 ^Z^
	NPFERT	5656 ^b^	10.36 ^yZ^	0.16 ^zY^	65.97 ^Z^	33.48 ^X^	62.82 ^Z^
2011	LOW	3932 ^cd^	7.90 ^zY^	0.08 ^yX^	70.78 ^Y^	34.11 ^X^	62.33 ^Z^
	DDGS	3316 ^d^	8.03 ^yzY^	0.09 ^yzX^	72.42 ^Y^	34.03 ^X^	62.39 ^Z^
	NFERT	3278 ^d^	9.71 ^yzY^	0.07 ^yX^	71.12 ^Y^	33.99 ^X^	62.42 ^Z^
	NPFERT	3560 ^d^	10.13 ^yY^	0.12 ^zX^	71.26 ^Y^	33.38 ^X^	62.89 ^Z^
2012	LOW	3013 ^d^	11.61 ^zX^	0.23 ^yZ^	82.68 ^W^	44.19 ^Z^	54.47 ^X^
	DDGS	2938 ^d^	12.74 ^yzX^	0.25 ^yzZ^	82.71 ^W^	42.83 ^Z^	55.53 ^X^
	NFERT	3053 ^d^	12.58 ^yzX^	0.25 ^yZ^	84.01 ^W^	43.47 ^Z^	55.04 ^X^
	NPFERT	3670 ^d^	13.30 ^yX^	0.24 ^zZ^	82.72 ^W^	43.52 ^Z^	55.00 ^X^
2013	LOW	6140 ^ab^	15.05 ^zW^	0.15 ^yY^	75.01 ^X^	39.68 ^Y^	57.99 ^Y^
	DDGS	5007 ^bc^	17.34 ^yzW^	0.17 ^yzY^	77.02 ^X^	39.91 ^Y^	57.81 ^Y^
	NFERT	6055 ^ab^	18.44 ^yzW^	0.14 ^yY^	75.38 ^X^	39.38 ^Y^	58.22 ^Y^
	NPFERT	7001^a^	18.45 ^yW^	0.24 ^zY^	74.61 ^X^	37.97 ^Y^	59.32 ^Y^
	SEM	485	0.93	0.02	0.91	1.11	0.86
*p*-value	TRT	0.01	0.01	0.03	0.01	0.08	0.08
	Year	0.07	0.01	0.01	0.01	0.01	0.01
	TRT*Year	0.04	0.77	0.36	0.36	0.78	0.78

^1^ TRT = treatment; CP = crude protein; P = phosphorus; NDF = neutral detergent fiber; ADF = acid detergent fiber; TDN = total digestible nutrients. ^2^ LOW = targeted stocking rate of 330 kg of BW/ha and no fertilizer or DDGS supplementation; DDGS = targeted stocking rate of 660 kg of BW/ha and no fertilizer with DDGS supplementation at a level of 0.75% of BW/day; NFERT = targeted stocking rate of 660 kg of BW/ha and 90 kg of N/ha with no DDGS supplementation; NPFERT = targeted stocking rate of 660 kg of BW/ha, 90 kg of N/ha, and 39 kg of P/ha with no DDGS supplementation. ^yz^ Means without a common superscript for effect of treatment differ at *p* ≤ 0.05. ^WXYZ^ Means without a common superscript for effect of year differ at *p* ≤ 0.05. ^abcd^ Means without a common superscript for effect of treatment × year interaction differ at *p* ≤ 0.05.

**Table 3:** Linear regression slopes for forage variables according to day of trial within a year for pastures grazed by stocker steers and heifers.

Year	TRT ^1^	Biomass (kg/ha/d)	CP (%/d)	P (%/d)	NDF (%/d)	ADF (%/d)	TDN (%/d)
2010	LOW ^2^	31.99 ^abc^	–0.026	–0.00044	0.032 ^ab^	0.034	–0.027
	DDGS	14.41 ^abc^	–0.010	–0.00002	–0.008 ^a^	0.013	–0.010
	NFERT	6.18 ^abc^	–0.042	–0.00032	0.013 ^a^	0.018	–0.014
	NPFERT	12.13 ^abc^	–0.057	–0.00014	0.074 ^abc^	0.068	–0.053
2011	LOW	–9.10 ^a^	–0.161	–0.00184	0.184 ^bcde^	0.133	–0.104
	DDGS	–30.25 ^a^	–0.164	–0.00209	0.251 ^de^	0.169	–0.131
	NFERT	–26.82 ^a^	–0.224	–0.00240	0.211 ^cde^	0.182	–0.142
	NPFERT	–35.09 ^a^	–0.250	–0.00320	0.263 ^e^	0.199	–0.155
2012	LOW	2.32 ^abc^	–0.032	0.00154	0.058 ^abc^	0.070	–0.054
	DDGS	–0.89 ^abc^	–0.007	0.00153	0.023 ^ab^	0.023	–0.018
	NFERT	–11.88 ^a^	–0.039	0.00099	0.071 ^abc^	0.110	–0.085
	NPFERT	–7.10 ^ab^	–0.022	0.00113	0.049 ^abc^	0.062	–0.048
2013	LOW	43.65^c^	0.055	–0.00049	0.066 ^abc^	0.060	–0.046
	DDGS	37.26 ^bc^	0.035	–0.00041	0.005 ^a^	0.015	–0.011
	NFERT	8.91 ^abc^	0.007	–0.00044	0.071 ^abc^	0.057	–0.045
	NPFERT	–11.36 ^a^	0.010	–0.00070	0.083 ^abcd^	0.056	–0.044
	SEM	11.82	0.022	0.00038	0.021	0.022	0.022
*p*-value	Day(Year)	0.01	0.01	0.01	0.01	0.01	0.01
	TRT*Day(Year)	0.01	0.61	0.97	0.04	0.24	0.24

^1^ TRT = treatment; CP = crude protein; P = phosphorus; NDF = neutral detergent fiber; ADF = acid detergent fiber; TDN = total digestible nutrients. ^2^ LOW = targeted stocking rate of 330 kg of BW/ha and no fertilizer or DDGS supplementation; DDGS = targeted stocking rate of 660 kg of BW/ha and no fertilizer with DDGS supplementation at a level of 0.75% of BW/day; NFERT = targeted stocking rate of 660 kg of BW/ha and 90 kg of N/ha with no DDGS supplementation; NPFERT = targeted stocking rate of 660 kg of BW/ha, 90 kg of N/ha, and 39 kg of P/ha with no DDGS supplementation. ^abcde^ Means without a common superscript for effect of treatment × day(year) interaction differ at *p* ≤ 0.05.

**Table 4:** Growth performance among treatments across 4 years in stocker steers and heifers.

Year	TRT ^1^	IBW (kg)	FBW (kg)	ADG (kg/d)	Gain (kg/ha)	Est. Forage DMI (kg/d)	Supp. DMI (kg/hd)	Forage Yield (kg/ha)
2010	LOW ^2^	279 ^Z^	408 ^yzZ^	0.97 ^abc^	162 ^zZ^	9.50 ^ab^	29.2 ^e^	15,447 ^yzY^
	DDGS	275 ^Z^	412 ^zZ^	1.03 ^ac^	339 ^yZ^	6.88 ^efg^	290.8 ^a^	12,019 ^zY^
	NFERT	272 ^Z^	386 ^yZ^	0.86 ^bcf^	262 ^xZ^	8.46 ^abcd^	29.2 ^e^	12,004 ^yzY^
	NPFERT	272 ^Z^	389 ^yZ^	0.88 ^bcf^	292 ^xZ^	8.66 ^abc^	29.2 ^e^	14,143 ^yY^
2011	LOW	238 ^Y^	307 ^yzY^	1.10 ^ad^	84 ^zY^	8.75 ^abc^	0.0 ^f^	7059 ^yzZ^
	DDGS	236 ^Y^	322 ^zY^	1.37 ^g^	222 ^yY^	6.55 ^efh^	136.8 ^d^	5323 ^zZ^
	NFERT	239 ^Y^	306 ^yY^	1.07 ^a^	166 ^xY^	8.61 ^abc^	0.0 ^f^	7464 ^yzZ^
	NPFERT	240 ^Y^	320 ^yY^	1.28 ^dg^	209 ^xY^	9.66 ^b^	0.0 ^f^	10,058 ^yZ^
2012	LOW	264 ^Z^	331 ^yzX^	0.56 ^e^	84 ^zY^	8.19 ^acd^	26.4 ^e^	-
	DDGS	266 ^Z^	355 ^zX^	0.75 ^fh^	218 ^yY^	5.63 ^eh^	235.4 ^b^	-
	NFERT	267 ^Z^	333 ^yX^	0.56 ^e^	158 ^xY^	8.12 ^cdg^	24.9 ^e^	-
	NPFERT	265 ^Z^	336 ^yX^	0.59 ^eh^	175 ^xY^	8.33 ^acd^	25.2 ^e^	-
2013	LOW	224 ^Y^	322 ^yzY^	0.82 ^bf^	139 ^zX^	8.24 ^acd^	29.5 ^e^	-
	DDGS	226 ^Y^	321 ^zY^	0.78 ^bf^	273 ^yX^	5.34 ^h^	203.2 ^c^	-
	NFERT	226 ^Y^	312 ^yY^	0.71 ^efh^	248 ^xX^	7.59 ^cdfg^	26.7 ^e^	-
	NPFERT	227 ^Y^	312 ^yY^	0.71 ^efh^	247 ^xX^	7.24 ^dfg^	27.3 ^e^	-
	SEM	9	7	0.03	17	0.29	2.6	1626
*p*-value	TRT	0.99	0.01	0.01	0.01	0.01	0.01	0.04
	Year	0.01	0.01	0.01	0.01	0.01	0.01	0.01
	TRT*Year	0.99	0.29	0.01	0.76	0.02	0.01	0.25

^1^ TRT = treatment; IBW = initial body weight; FBW = final body weight; ADG = average daily gain; Supp = supplement. ^2^ LOW = targeted stocking rate of 330 kg of BW/ha and no fertilizer or DDGS supplementation; DDGS = targeted stocking rate of 660 kg of BW/ha and no fertilizer with DDGS supplementation at a level of 0.75% of BW/day; NFERT = targeted stocking rate of 660 kg of BW/ha and 90 kg of N/ha with no DDGS supplementation; NPFERT = targeted stocking rate of 660 kg of BW/ha, 90 kg of N/ha, and 39 kg of P/ha with no DDGS supplementation. ^xyz^ Means without common superscripts for effect of treatment differ at *p* ≤ 0.05. ^XYZ^ Means without common superscripts for effect of year differ at *p* ≤ 0.05. ^abcdefgh^ Means without common superscripts for effect of treatment × year interaction differ at *p* ≤ 0.05.

**Table 5:** Income, expenses, and returns among treatments across 4 years in stocker steers and heifers.

Year	TRT ^1^	Feed Cost (USD/hd)	Fertilizer Cost (USD/hd)	Variable Input Cost (USD/hd)	Revenue (USD/hd)	Returns (USD/hd)	Returns (USD/ha)
2010	LOW	9.39 ^d^	0.00 ^f^	9.39 ^hi^	865 ^yzW^	293.50 ^yX^	370.30 ^ef^
	DDGS	37.60 ^b^	0.00 ^f^	37.60 ^g^	870 ^yW^	270.20 ^yX^	670.90 ^ab^
	NFERT	9.39 ^d^	40.50 ^e^	49.89 ^ef^	842 ^zW^	229.90 ^zX^	548.80 ^bcd^
	NPFERT	9.39 ^d^	57.60 ^cd^	67.01 ^c^	845 ^yzW^	215.80 ^zX^	539.50 ^cd^
2011	LOW	0.00 ^e^	0.00 ^f^	0.00 ^i^	899 ^yzX^	80.90 ^yY^	96.00 ^h^
	DDGS	36.06 ^b^	0.00 ^f^	36.06 ^g^	938 ^yX^	66.30 ^yY^	166.70 ^gh^
	NFERT	0.00 ^e^	44.10 ^e^	44.12 ^fg^	898 ^zX^	36.00 ^zY^	92.40 ^h^
	NPFERT	0.00 ^e^	69.30 ^b^	69.30 ^bc^	934 ^yzX^	46.50 ^zY^	122.40 ^h^
2012	LOW	9.91 ^cd^	0.00 ^f^	9.91 ^hi^	1037 ^yzY^	118.20 ^yZ^	149.20 ^h^
	DDGS	61.84 ^a^	0.00 ^f^	61.84 ^cd^	1087 ^yY^	115.40 ^yZ^	282.90 ^fg^
	NFERT	9.36 ^d^	56.20 ^d^	65.55 ^cd^	1043 ^zY^	67.90 ^zZ^	167.10 ^gh^
	NPFERT	9.46 ^d^	81.80 ^a^	91.22 ^a^	1048 ^yzY^	47.90 ^zZ^	118.60 ^h^
2013	LOW	13.91 ^c^	0.00 ^f^	13.91 ^h^	1116 ^yzZ^	321.00 ^yX^	443.50 ^de^
	DDGS	60.85 ^a^	0.00 ^f^	60.85 ^cd^	1112 ^yZ^	270.00 ^yX^	761.70 ^a^
	NFERT	12.61 ^cd^	43.30 ^e^	55.93 ^de^	1091 ^zZ^	254.70 ^zX^	743.60 ^a^
	NPFERT	12.87 ^cd^	66.50 ^bc^	79.33 ^b^	1092 ^yzZ^	231.70 ^zX^	652.40 ^abc^
	SEM	0.77	1.78	1.96	15.7	15.10	23.80
*p*-value	TRT	0.01	0.01	0.01	0.03	0.01	0.01
	Year	0.01	0.01	0.01	0.01	0.01	0.01
	TRT*Year	0.01	0.01	0.01	0.56	0.65	0.01

^1^ TRT = treatment; LOW = targeted stocking rate of 330 kg of BW/ha and no fertilizer or DDGS supplementation; DDGS = targeted stocking rate of 660 kg of BW/ha and no fertilizer with DDGS supplementation at a level of 0.75% of BW/day; NFERT = targeted stocking rate of 660 kg of BW/ha and 90 kg of N/ha with no DDGS supplementation; NPFERT = targeted stocking rate of 660 kg of BW/ha, 90 kg of N/ha, and 39 kg of P/ha with no DDGS supplementation. ^yz^ Means without common superscripts for effect of treatment differ at *p* ≤ 0.05. ^WXYZ^ Means without common superscripts for effect of year differ at *p* ≤ 0.05. ^abcdefghi^ Means without common superscripts for effect of treatment × year interaction differ at *p* ≤ 0.05.

**Table 6:** Sensitivity analysis of dried distiller’s grains and urea fertilizer price on difference in returns (USD/hd) between the DDGS and NFERT management systems.

Urea	Dried Distiller’s Grains						
	–30% ^1^	–20%	–10%	0%	+10%	+20%	+30%
−30%	36.72	30.87	25.02	19.17	13.32	7.47	1.62
−20%	41.52	35.67	29.82	23.97	18.12	12.27	6.42
−10%	46.32	40.47	34.62	28.77	22.92	17.07	11.22
0%	51.12	45.27	39.42	33.57	27.72	21.87	16.02
+10%	55.92	50.07	44.22	38.37	32.52	26.67	20.82
+20%	60.72	54.87	49.02	43.17	37.32	31.47	25.62
+30%	65.52	59.67	53.82	47.97	42.12	36.27	30.42

^1^ Percentage change in the base price of dried distiller’s grains (USD 215/Mg) and urea fertilizer (USD 608/Mg).

**Table 7:** Nitrogen and phosphorus use efficiency among treatments across 4 years in stocker steers and heifers.

Year	TRT ^1^	N Inputs (kg/ha)	N Retained (kg/ha)	NUE (%)	P Inputs (kg/ha)	P Retained (kg/ha)	PUE (%)
2010	LOW ^2^	5.74 ^a^	3.90 ^zZ^	67.90 ^a^	3.50 ^ab^	1.10 ^zZ^	38.39 ^z^
	DDGS	38.38 ^c^	8.10 ^xZ^	21.10 ^de^	71.38 ^c^	2.28 ^xZ^	3.22 ^y^
	NFERT	97.89 ^f^	6.50 ^yZ^	6.64 ^f^	7.11 ^ab^	1.83 ^yZ^	27.37 ^z^
	NPFERT	98.30 ^fh^	7.22 ^xyZ^	7.34 ^ef^	42.45 ^gh^	2.03 ^xyZ^	4.79 ^y^
2011	LOW	3.17 ^b^	2.27 ^zY^	71.78 ^a^	0.00 ^a^	0.64 ^zY^	-
	DDGS	19.77 ^d^	5.88 ^xY^	29.74 ^cd^	30.61 ^d^	1.65 ^xY^	5.46 ^y^
	NFERT	93.17 ^g^	4.52 ^yY^	4.85 ^f^	0.00 ^a^	1.27 ^yY^	-
	NPFERT	93.17 ^g^	5.56 ^xyY^	5.97 ^f^	35.00 ^dg^	1.56 ^xyY^	4.47 ^y^
2012	LOW	6.02 ^a^	2.34 ^zY^	38.90 ^bc^	3.21 ^ab^	0.66 ^zY^	23.47 ^z^
	DDGS	35.05 ^e^	5.89 ^xY^	16.81 ^def^	52.05 ^e^	1.66 ^xY^	3.20 ^y^
	NFERT	97.77 ^f^	4.38 ^yY^	4.48 ^f^	6.56 ^ab^	1.23 ^yY^	20.10 ^z^
	NPFERT	97.97 ^f^	4.83 ^xyY^	4.93 ^f^	41.65 ^gh^	1.36 ^xyY^	3.26 ^y^
2013	LOW	7.32 ^a^	3.86 ^zZ^	52.84 ^b^	4.03 ^ab^	1.09 ^zZ^	29.42 ^z^
	DDGS	33.27 ^e^	7.62 ^xZ^	22.76 ^d^	61.53 ^f^	2.14 ^xZ^	3.46 ^y^
	NFERT	99.99 ^h^	6.99 ^yZ^	6.98 ^ef^	8.57 ^b^	1.97 ^yZ^	23.74 ^z^
	NPFERT	100.10 ^h^	6.99 ^xyZ^	6.98 ^ef^	43.57 ^h^	1.97 ^xyZ^	4.46 ^y^
	SEM	0.39	0.51	2.69	2.07	0.14	4.63
*p*-value	TRT	0.01	0.01	0.01	0.01	0.01	0.01
	Year	0.01	0.01	0.01	0.01	0.01	0.23
	TRT*Year	0.01	0.95	0.01	0.01	0.95	0.71

^1^ TRT = treatment; N = nitrogen; NUE = nitrogen use efficiency (retained/inputs); P = phosphorus; PUE = phosphorus use efficiency (retained/inputs) ^2^ LOW = targeted stocking rate of 330 kg of BW/ha and no fertilizer or DDGS supplementation; DDGS = targeted stocking rate of 660 kg of BW/ha and no fertilizer, with DDGS supplementation at a level of 0.75% of BW/day; NFERT = targeted stocking rate of 660 kg of BW/ha and 90 kg of N/ha with no DDGS supplementation; NPFERT = targeted stocking rate of 660 kg of BW/ha, 90 kg of N/ha, and 39 kg of P/ha with no DDGS supplementation. ^xyz^ Means without common superscripts for effect of treatment differ at *p* ≤ 0.05. ^YZ^ Means without common superscripts for effect of year differ at *p* ≤ 0.05. ^abcdefgh^ Means without common superscripts for effect of treatment × year interaction differ at *p* ≤ 0.05.

**Table 8:** Estimated greenhouse gas emissions among treatments across 4 years in stocker steers and heifers.

Year	TRT ^1^	Enteric CH_4_ (kg)	Manure CH_4_ (kg)	Manure N_2_O (kg)	Fertilizer N_2_O (kg)	Direct Emissions (kg CO_2_eq)	Indirect Emissions (kg CO_2_eq)	Total Emissions (kg CO_2_eq)	Total Emissions (kg CO_2_eq/kg gain)
2010	LOW ^2^	563 ^zY^	12.2 ^zZ^	5.66 ^ab^	0.0 ^z^	16,069 ^zY^	162 ^z^	16,231 ^zY^	12.48 ^zZ^
	DDGS	1215 ^yY^	24.4 ^yZ^	18.12 ^efg^	0.0 ^z^	36,385 ^yY^	4776 ^y^	41,160 ^yY^	12.93 ^zZ^
	NFERT	1039 ^yY^	22.3 ^yZ^	11.70 ^bcdf^	12.6 ^y^	33,780 ^yY^	6625 ^x^	40,405 ^yY^	17.08 ^yZ^
	NPFERT	1128 ^yY^	24.5 ^yZ^	12.70 ^cdf^	12.4 ^y^	36,292 ^yY^	6728 ^x^	43,020 ^yY^	16.69 ^yZ^
2011	LOW	223 ^zX^	4.9 ^zY^	1.52 ^a^	0.0 ^z^	6152 ^zZ^	0 ^z^	6152 ^zZ^	9.49 ^zY^
	DDGS	569 ^yX^	11.1 ^yY^	7.13 ^abcd^	0.0 ^z^	16,634 ^yZ^	2230 ^y^	18,955 ^yZ^	9.16 ^zY^
	NFERT	516 ^yX^	11.4 ^yY^	4.61 ^a^	12.6 ^y^	18,314 ^yZ^	6295 ^x^	24,609 ^yZ^	16.62 ^yY^
	NPFERT	584 ^yX^	12.7 ^yY^	5.39 ^ab^	12.4 ^y^	20,208 ^yZ^	6383 ^x^	26,591 ^yZ^	14.78 ^yY^
2012	LOW	441 ^zZ^	11.4 ^zZ^	5.85 ^abc^	0.0 ^z^	13,041 ^zY^	142 ^z^	13,182 ^zY^	19.41 ^zX^
	DDGS	936 ^yZ^	20.7 ^yZ^	18.13 ^efg^	0.0 ^z^	29,322 ^yY^	3830 ^y^	33,152 ^yY^	16.24 ^zX^
	NFERT	902 ^yZ^	23.1 ^yZ^	12.89 ^df^	12.6 ^y^	30,735 ^yY^	6585 ^x^	37,319 ^yY^	26.55 ^yX^
	NPFERT	948 ^yZ^	24.3 ^yZ^	13.98 ^dfg^	12.4 ^y^	32,164 ^yY^	6676 ^x^	38,840 ^yY^	26.45 ^yX^
2013	LOW	511 ^zYZ^	12.3 ^zZ^	8.21 ^abcd^	0.0 ^z^	15,527 ^zY^	180 ^z^	15,707 ^zY^	13.96 ^zW^
	DDGS	987 ^yYZ^	21.2 ^yZ^	21.50 ^e^	0.0 ^z^	31,611 ^yY^	3884 ^y^	35,495 ^yY^	14.00 ^zW^
	NFERT	1036 ^yYZ^	24.7 ^yZ^	21.21 ^e^	12.6 ^y^	36,601 ^yY^	6677 ^x^	43,279 ^yY^	20.06 ^yW^
	NPFERT	995 ^yYZ^	23.2 ^yZ^	19.89 ^eg^	12.4 ^y^	35,081 ^yY^	6765 ^x^	41,845 ^yY^	19.89 ^yW^
	SEM	137	3.1	2.07	1.4	4463	785	5172	1.08
*p*-value	TRT	0.01	0.01	0.01	0.01	0.01	0.01	0.01	0.01
	Year	0.01	0.01	0.01	1.00	0.01	0.18	0.01	0.01
	TRT*Year	0.78	0.73	0.02	1.00	0.75	0.70	0.76	0.13

^1^ TRT = treatment; CH_4_ = methane; N_2_O = nitrous oxide; CO_2_ = carbon dioxide. ^2^ LOW = targeted stocking rate of 330 kg of BW/ha and no fertilizer or DDGS supplementation; DDGS = targeted stocking rate of 660 kg of BW/ha and no fertilizer with DDGS supplementation at a level of 0.75% of BW/day; NFERT = targeted stocking rate of 660 kg of BW/ha and 90 kg of N/ha with no DDGS supplementation; NPFERT = targeted stocking rate of 660 kg of BW/ha, 90 kg of N/ha, and 39 kg of P/ha with no DDGS supplementation. ^xyz^ Means witout common superscripts for effect of treatment differ at *p* ≤ 0.05. ^WXYZ^ Means without common superscripts for effect of year differ at *p* ≤ 0.05. ^abcdefg^ Means without common superscripts for effect of treatment × year interaction differ at *p* ≤ 0.05.

## Data Availability

The data presented in this study are available upon request from the corresponding author.
